# Comparative pan-genomic analysis reveals pathogenic mechanisms and genomic plasticity in *Vibrio parahaemolyticus* clinical and environmental isolates

**DOI:** 10.3389/fcimb.2025.1574627

**Published:** 2025-04-10

**Authors:** Peng Zhang, Xiaofang Wu, Lei Ji, Wei Yan, Liping Chen, Fenfen Dong

**Affiliations:** Microbiology Laboratory, Huzhou Center for Disease Control and Prevention, Huzhou, Zhejiang, China

**Keywords:** *Vibrio parahaemolyticus*, pan-genome, mobile genetic element, pathogenic characteristic, genomic plasticity

## Abstract

**Introduction:**

*Vibrio parahaemolyticus* is a human pathogen capable of inducing bacterial gastroenteritis. Clinical strains of *V. parahaemolyticus* are considered pathogenic due to their possession of hemolysin and a type III secretion system (T3SS). Some environmental isolates are also acquiring corresponding virulence genes.

**Methods:**

This study initially examines the infection characteristics of *V. parahaemolyticus*, and subsequently employs pan-genomic analysis to identify genes that exhibit significant differences in distribution between environmental and clinical isolates, thereby revealing their potential impact on virulence.

**Results and discussion:**

The epidemiological analysis of clinical isolates suggests that infections of *V. parahaemolyticus* are more prevalent in warm seasons, with O4:KUT serotype presenting more severe symptoms. OrthoFinder analysis revealed that environmental isolates possess a higher number of core genes. PEPPAN and KEGG analysis revealed that the 10 genes exclusively found in clinical isolates were predominantly associated with virulence. Additionally, the functions of genes differentially distributed in the environment were significantly more diverse compared to those in clinical settings. Analysis of mobile genetic elements suggested that environmental isolates harbor more mobile genetic elements, implying a potential for an increased number of resistance genes. The pathogenic characteristics of the strains examined in this study, genomic diversity and variation in mobile genetic elements are highly significant for deepening our understanding of the pathogenic mechanisms of *V. parahaemolyticus* and for the development of strategies to prevent its infections.

## 1 Introduction

*Vibrio parahaemolyticus*, commonly found in estuarine and marine environments globally, is recognized as one of the most prevalent foodborne pathogens (Letchumanan et al., 2014). Consumption of raw or undercooked seafood can result in acute gastroenteritis, wound infections, and septicemia (Newton et al., 2012). While gastroenteritis may be self-limiting, infections can progress to sepsis, posing a life-threatening risk for individuals with a history of the disease (Letchumanan et al., 2014).

*V. parahaemolyticus* is classified through serotyping, which is determined by the combination of somatic (O) and capsular (K) antigens. Specifically, *V. parahaemolyticus* can be categorized into 13 O-antigen and 71 K-antigen types (Iguchi et al., 1995). In February 1996, a food poisoning outbreak caused by a novel O3:K6 serotype emerged in India, which rapidly spread and evolved into a pandemic clone (Okuda et al., 1997). Following the 1996 outbreak of the O3:K6 strain, *V. parahaemolyticus* has increasingly become one of the leading foodborne pathogens worldwide (Hara-Kudo et al., 2012). The O4:KUT serotype first appeared in Zhejiang Province in 2013 and quickly became the predominant serotype that year (Zhu et al., 2020). Since 2017, *V. parahaemolyticus* O4:KUT has remained the dominant serotype. However, in 2020, the O10:K4 serotype was detected for the first time in Huzhou City, eventually surpassing O3:K6 to become the new dominant serotype (Zhang et al., 2022). Similar trends have been observed in other regions, including Beijing (Huang et al., 2022b), Guangzhou (He et al., 2022), and Guangxi (Huang et al., 2022a). Notably, Thailand reported its first case of O10:K4 infection in 2021, isolated from an inpatient with acute diarrhea, confirming the emergence of the O10:K4 serotype in Southeast Asia (Okada et al., 2023).

*V. parahaemolyticus* is naturally prevalent in marine environments. However, recent observations indicate a rise in the incidence of *V. parahaemolyticus* in inland cities across China, potentially attributable to the contamination of freshwater fish with this bacterium (Lei et al., 2020). Over the past few years, *V. parahaemolyticus* has been isolated from various freshwater food sources, including crayfish, fish, shrimp, and sediments (Jiang et al., 2020; Chen et al., 2021). The majority of *V. parahaemolyticus* strains isolated from environmental sources are typically non-pathogenic, in contrast to clinical isolates, which often represent pathogenic clones. Pathogenic strains of *V. parahaemolyticus* are known to harbor virulence factors, including the genes encoding thermostable direct hemolysin (*tdh*) and tdh-related hemolysin (*trh*), whereas most environmental isolates lack these virulence genes (Martinez-Urtaza et al., 2008; Flores-Primo et al., 2014; Haley et al., 2014; Letchumanan et al., 2015). Our previous studies have demonstrated significant differences in the serotypes and virulence genes between clinical and environmental isolates of *V. parahaemolyticus* (Zhang et al., 2024).

The objective of this study is to explore the genomic characteristics of pathogenic *V. parahaemolyticus* isolates from both clinical and environmental sources, analyze the differences and relationships between them, and elucidate the underlying factors contributing to the pathogenicity of clinical isolates. We collected 69 clinical isolates from patients and 59 isolates from environment. Employing pan-genomic approach, we identified distinctive features among genomes categorized by host origin. Our preliminary analysis has delineated genomic differences between clinical and environmental isolates, shedding light on the genetic basis of pathogenicity in clinical isolates.

## 2 Materials and methods

### 2.1 Bacterial isolate

The sequencing data have been deposited in the NCBI database under the BioProject accession number PRJNA1071824. Detailed information regarding the isolate has been presented in our previous article (Zhang et al., 2024). Metadata including gender, patient age, hospitalization status and main symptoms were collected. Mobile genetic elements, including plasmids and insertion sequences (IS), were identified using the online software MobileElementFinder version 1.0.3, which is accessible at https://cge.food.dtu.dk/services/MobileElementFinder/ (Johansson et al., 2021). PhiSpy (Akhter et al., 2012) was used with its default parameters, employing GBK files (GenBank files) annotated by PROKKA (Seemann, 2014) as input to predict the presence of prophages. The histogram was generated using KaleidaGraph version 4.5.0. Multi-categorical alluvial diagrams were generated using RawGraphs 2.0 (Mauri et al., 2017). Virulence factors were predicted from the assembled genomes of each isolate using the Virulence Factor Database (VFDB) VFanalyzer tool, available at http://www.mgc.ac.cn/cgi-bin/VFs/v5/main.cgi?func=VFanalyzer. The heatmap was generated using the “pheatmap” package in R software.

### 2.2 Comparative genomics and phylogenetic analysis

The whole-genome DNA sequences from all samples were converted into protein sequences using gffread version 0.12.7 (Pertea and Pertea, 2020). Subsequently, these protein sequences were clustered with OrthoFinder version 2.5.5 (Emms and Kelly, 2019). The bar chart and associated plots were generated using KaleidaGraph version 4.5.0.

### 2.3 Pangenome analysis

Assembled contigs’ FASTA files were initially annotated using PROKKA (Seemann, 2014). For the pangenome analysis, PEPPAN (Zhou et al., 2020) was employed with the default settings, utilizing the GFF3 files annotated by PROKKA as inputs for the pipeline. Subsequently, the PEPPAN_parser script was applied to generate the final output. We comparatively analyzed each identified gene from clinical and environmental sources. Statistical analysis was performed using the chi-square test or Fisher’s exact test. Given the extensive number of genes, we focused our analysis on those with a *p*-value less than 0.01, indicating significant differences in distribution. To clarify the distribution patterns, we use the following notation: C-Yes to indicate the presence of a gene in clinical isolates, C-No to denote its absence in clinical isolates, E-Yes to represent its presence in environmental isolates, and E-No to signify its absence in environmental isolates. Those genes exhibiting significant differences in distribution underwent COG (Cluster of Orthologous Groups) annotation using the eggNOG 4.5 server (Huerta-Cepas et al., 2016). Pathway analysis of enriched COG classes was explored using the Kyoto Encyclopedia of Genes and Genomes (KEGG) pathway database (Kanehisa and Goto, 2000). The R packages “dotplot” and “clusterProfile” were used to analyze and visualize the KEGG analysis. The R packages “ggplot “ and “clusterProfile” were used to analyze and visualize the Gene Ontology (GO) analysis.

### 2.4 Phylogenetic analysis

We constructed a phylogenetic tree using methods described in previous literature (Zhang et al., 2024). A phylogenetic tree was constructed based on 69 single nucleotide polymorphisms (SNPs) identified among clinical isolates. Variant calling was performed against the *V. parahaemolyticus* RIMD 2210633 (GCF_000196095.1) genome using the Snippy pipeline (https://github.com/tseemann/snippy). Core SNP alignment was generated using snippy-core v.4.6.0, and recombinant regions were filtered out from the alignment using Gubbins v.3.0.0 (Croucher et al., 2015). SNPs were subsequently extracted from the alignment using SNP-sites (Page et al., 2016). The core SNP alignment was used to infer a phylogenetic tree with IQ-TREE, employing the maximum likelihood method and the TVMe+ASC nucleotide substitution model (Kalyaanamoorthy et al., 2017; Minh et al., 2020). Finally, the tree was visualized using FigTree v.1.4.4. We employed the snp-dists v0.8.2 software to convert FASTA format files into a matrix of SNP distances.

### 2.5 Statistical analysis

Statistical processing was performed using either the chi-square test or Fisher’s exact test in R software. The test selection depended on sample size and occurrence frequency. Chi-square test was used for samples ≥40 with frequencies ≥5. Fisher’s exact test was applied for smaller samples or frequencies ≤5. The Odds Ratio (OR) was used to assess the distribution patterns of each gene between clinical and environmental isolates.

## 3 Results

### 3.1 Patient characteristics

**Figure 1:**
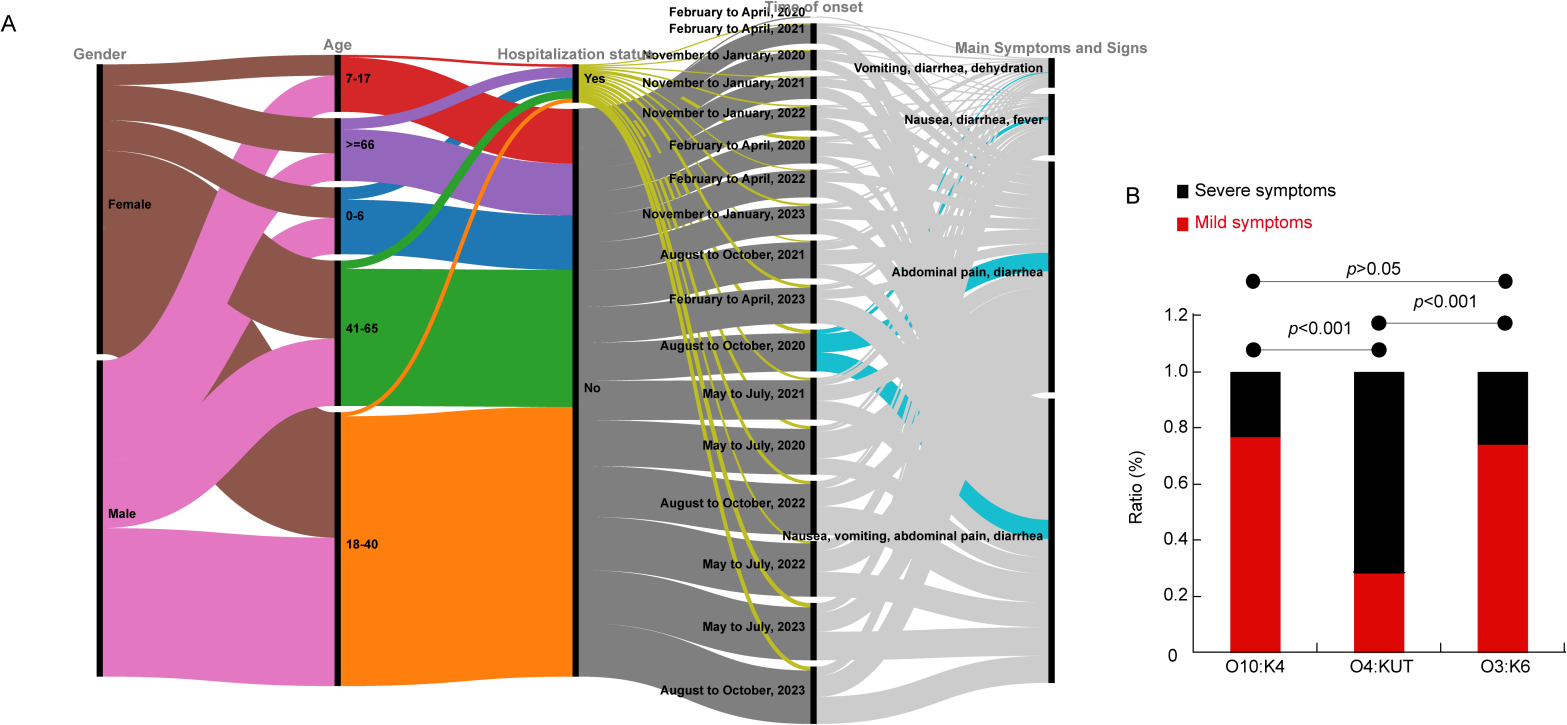
Characteristics of clinical patients. **(A)** The gender, age, hospitalization status, time of onset and main symptoms of the patients are represented in a multi-categorical alluvial diagram. **(B)** Bar chart showing symptom variations among different serotypes.

To study the characteristics of patients infected with *V. parahaemolyticus*, we collected metadata from 2020 to 2023 for patients with diarrhea. The data included gender, age, hospitalization status, main symptoms, and onset time, and was gathered from a total of 9,413 patients. Among these patients analyzed, the infection rates were 3.07% (151/4,911) in male patients and 3.53% (159/4,502) in female patients. Statistical analysis revealed no significant gender-based difference in *V. parahaemolyticus* infection rates (*p* = 0.24). The age group most affected was 18-40 years old, followed by those aged 41-65. The majority of patients did not require hospitalization; however, 6% of the patients needed hospital care. Hospitalized patients were found across all age groups. The primary symptoms reported were nausea, vomiting, abdominal pain, and diarrhea. In severe cases, patients also experienced fever and dehydration (**Figure 1A**). We categorize symptoms such as dehydration, fatigue, and fever as systemic and more severe, while considering digestive system symptoms like abdominal pain and diarrhea to be milder in comparison. We compared the clinical characterizations among patients infected with O10:K4, O3:K6, and O4:KUT serotypes and discovered that the phenotype associated with O4:KUT was significantly more severe than those associated with O10:K4 and O3:K6 (**Figure 1B**). The months with the highest incidence of illness were May to October (**Figure 1A**).

### 3.2 Evolution of clinical isolates and patient symptoms analysis

**Figure 2:**
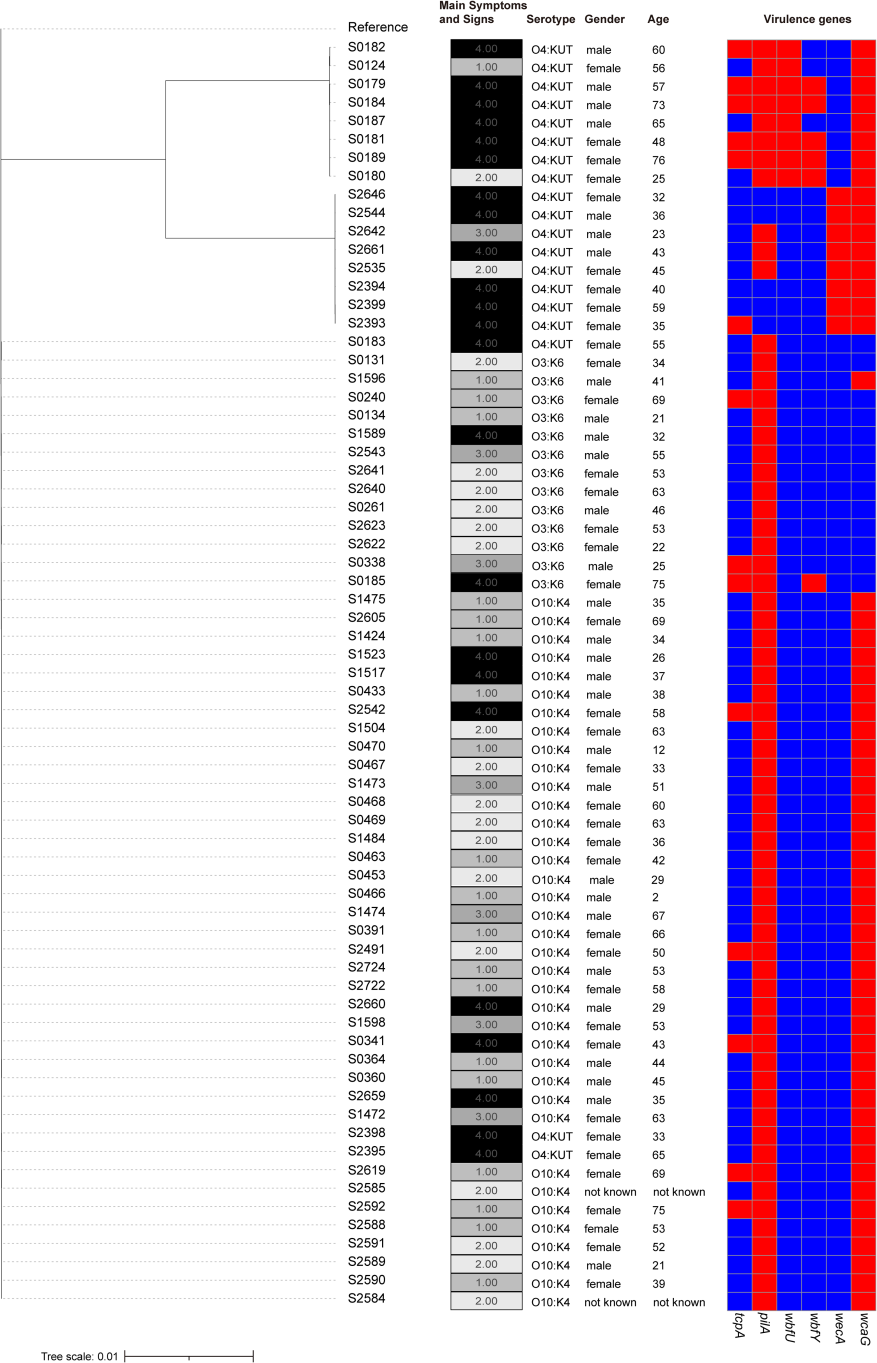
Phylogenetic tree of clinical isolates. A phylogenetic tree depicting the genetic relationships among 69 clinical isolates, adjacent columns on the right detail serotypes, symptom profiles, gender, age of patients and virulence genes for each isolate. patients were categorized into four severity levels based on clinical manifestations: level 1 for abdominal pain and diarrhea, level 2 for abdominal pain, diarrhea, and vomiting, level 3 for abdominal pain, diarrhea, vomiting, and fever, and level 4 for the additional presence of thirst and fatigue. In the heatmap, the presence of virulence genes is indicated by red, while their absence is represented by blue.

To further investigate the pathogenicity of clinical isolates, we utilized the sequenced isolates for additional analysis. Initially, we constructed an evolutionary tree for the clinical isolates and observed that a cluster of O4:KUT demonstrated increased phylogenetic distance compared to other isolates (**Figure 2**; **Supplementary Table S1**). This O4:KUT cluster was further divided into two distinct branches (**Figure 2**). While there was minor variation in the number of SNPs within each O4:KUT branch, significant differences were observed between branches. Additionally, substantial SNP differences exist between O4:KUT and other serotypes (**Supplementary Table S1**). To gain a clearer understanding of the patient’s clinical symptoms, we have categorized them into four severity levels: level 1 for abdominal pain and diarrhea, level 2 for abdominal pain, diarrhea, and vomiting, level 3 for abdominal pain, diarrhea, vomiting, and fever, and level 4 for the additional presence of thirst and fatigue. We found that the majority of patients with severe symptoms were infected with the O4:KUT serotype. The disease infection was not associated with gender (**Figure 2**). To investigate the underlying reasons for the more severe clinical symptoms associated with the O4:KUT serotype compared to O3:K6 and O10:K4, we predicted the virulence genes in these clinical isolates. For clarity, we focused only on virulence genes that exhibited differential distribution patterns across the isolates, excluding those with uniform distribution. Our analysis revealed differences in the distribution of six virulence genes, *tcpA*, *wbfU*, *wbfY* and *wecA* were predominantly found in the O4:KUT serotype (**Figure 2**).

### 3.3 Orthologs identification

**Figure 3:**
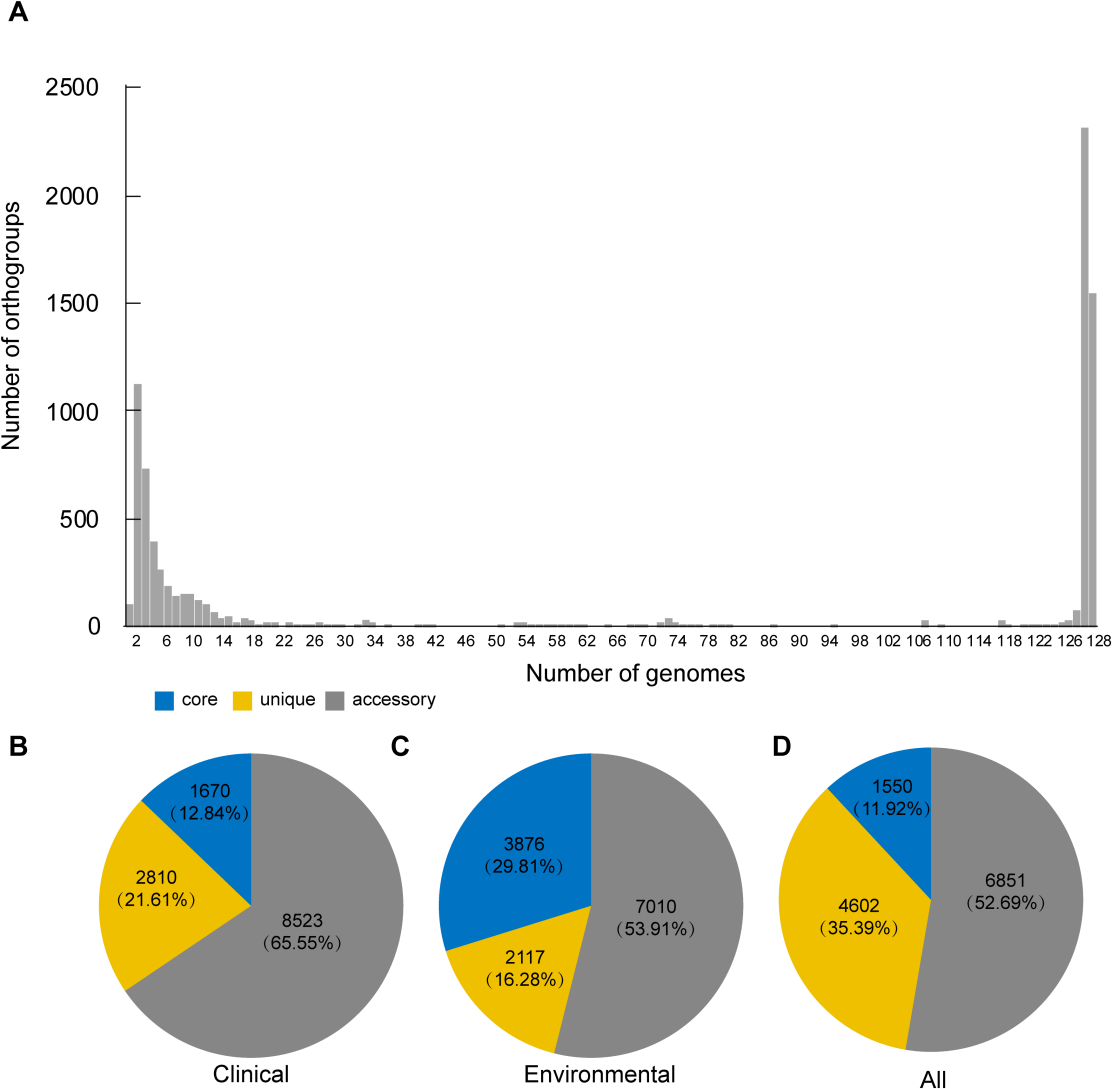
Overall orthogroup statistics and comparison. **(A)** Bar plot showing the number of orthologous clusters. **(B–D)** Summary of core, unique, and accessory orthogroups in the genomes of clinical **(B)**, environmental **(C)** and all isolates **(D)**. The numbers indicate the total counts and percentages of each orthogroup category.

Next, we intended to investigate the genomic differences between clinical and environmental isolates. To identify orthologous groups within the coding genes of both clinical and environmental isolates, we submitted 128 samples to OrthoFinder to identify orthologous groups. Our objective was to ascertain the distribution of gene families among these isolates. As shown in **Figure 3A**, the majority of non-core gene families are not broadly distributed. Furthermore, we investigated the differences in the distribution of these orthogroups between clinical and environmental isolates. OrthoFinder identified 13,003 orthogroups in both clinical and environmental isolates. We determined that 12.84% (1,670) of these orthologous groups were exclusively present in all clinical isolates, while 29.81% (3,876) were exclusively present in all environmental isolates. Furthermore, we observed that 65.55% (8,523) of the orthologous groups were present in at least two clinical isolates, while 53.91% (7,010) were found in at least two environmental isolates. Additionally, 21.61% (2,810) of the orthologous groups were unique to all clinical isolates, and 16.28% (2,117) were unique to all environmental isolates. In our analysis of the 128 isolates, we identified core orthogroups comprising 11.92% (1,550) of the total, unique orthogroups accounting for 35.39% (4,602), and accessory orthogroups representing 52.69% (6,851) (**Figures 3B–D**).

### 3.4 Pan-genomic analysis

**Figure 4:**
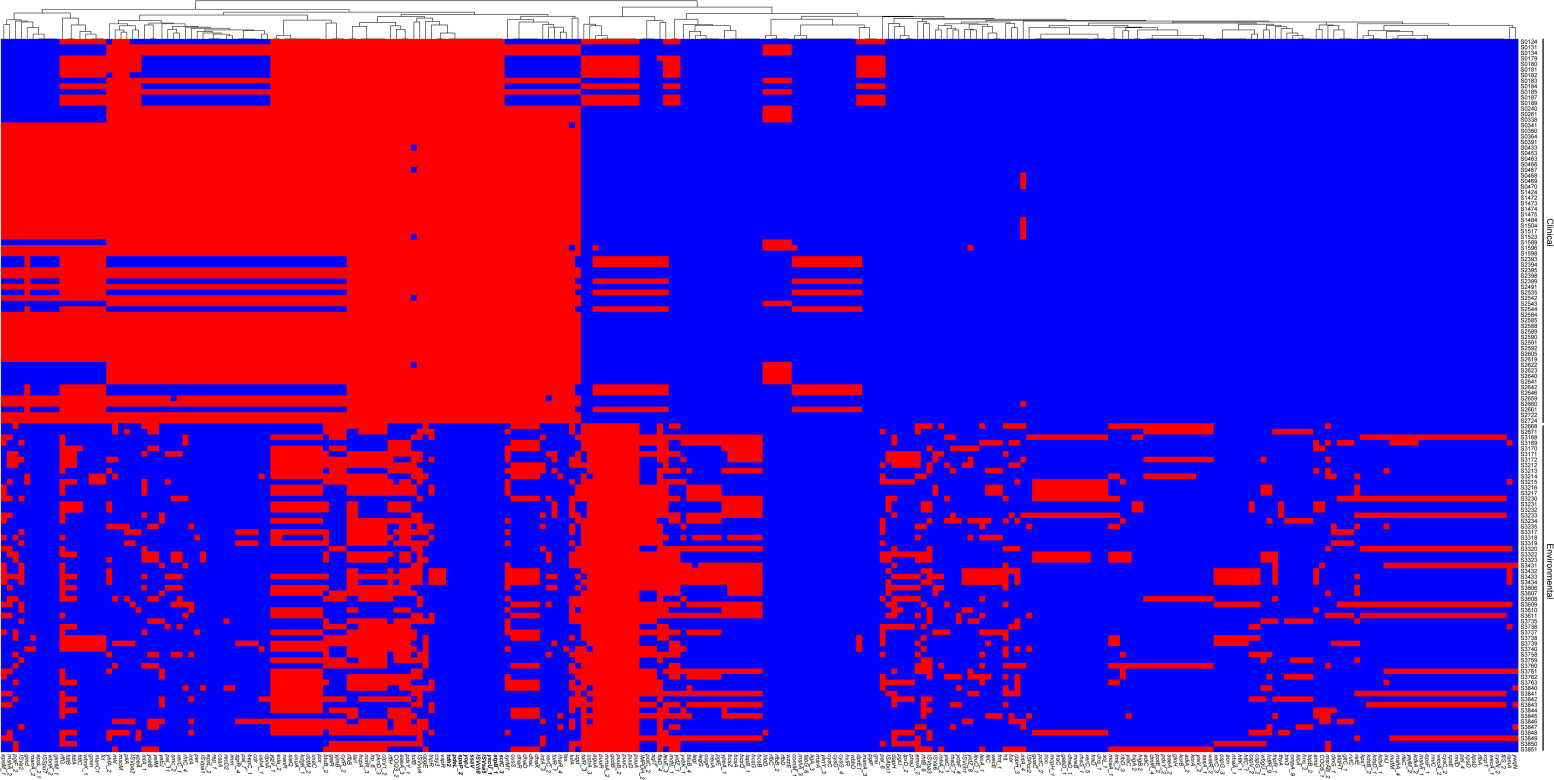
Heatmap displaying the pan-genome genes across 128 strains. Strain labels were located at the bottom, and pan-genome gene clusters were indicated on the left. The presence of genes was indicated by red color, while absence was denoted by blue.

**Figure 5:**
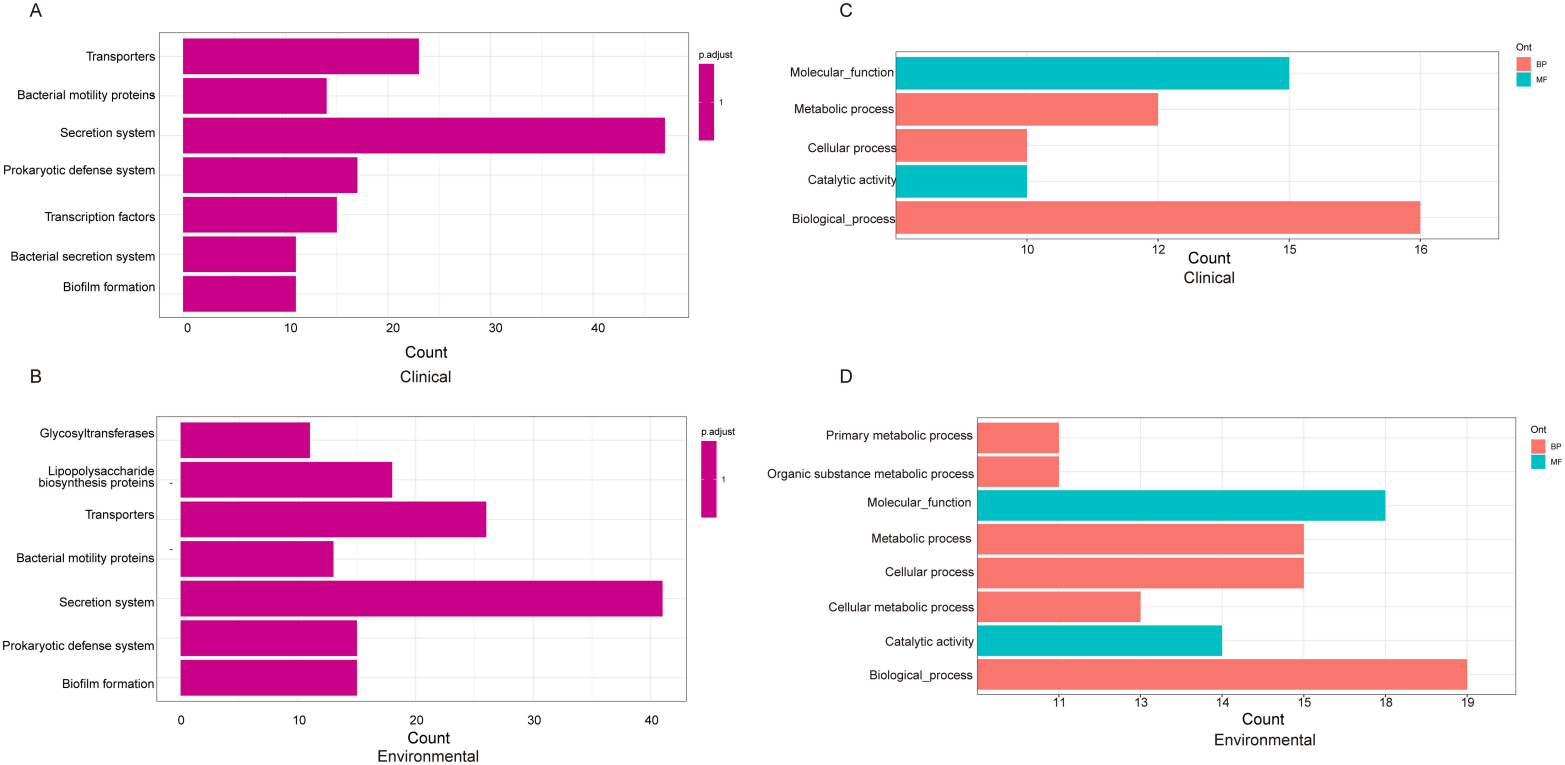
KEGG pathway analysis depicting the distribution of genes from **Figure 4** among clinical **(A)** and environmental strains **(B)**. The COG (Clusters of Orthologous Groups) classification and distribution of genes in clinical **(C)** and environmental strains **(D)**.

Following our analysis, we sought to explore the differences between clinical and environmental isolates at the individual gene level. For this pan-genomic analysis, we employed the software PEPPAN. The PEPPAN output revealed a total of 15,783 genes among all isolates. The strict core gene set, present in 100% of the sequences, comprised 3,712 genes. Additionally, 83 genes were identified as core genes present in 99%-100% of the sequences. The soft core gene category, shared by 95%-99% of the isolates, included 102 genes. The accessory gene set, also known as the shell genes, which are shared by 15% to less than 95% of the strains, contained 875 genes. Conversely, the cloud genes, found in 0% to less than 15% of the strains, numbered 11,011 in total (**Supplementary Figure S1**). A total of 1,677 genes showed statistically significant differences in their distribution patterns between clinical and environmental isolates (**Supplementary Table S2**). **Figure 4** displays the distribution of 259 genes with distinct names. Among the 259 genes identified, 120 were found predominantly in clinical isolates, while 139 were primarily detected in environmental isolates (**Supplementary Table S3**). We found that 10 genes were exclusively distributed in clinical isolates, and these genes were identified as *atpB*, *sctC*, *yscU*, *ISVsa5*, *ISVch8*, *ssaV*, *yopJ*, *spaP*, *pdeL* and *tdh2*. Among the 1677 genes examined, only 464 genes corresponded to the KO (KEGG Orthology) numbers. Of these, 226 genes showed a distribution bias toward clinical isolates, while 238 genes were more prevalent in environmental isolates (**Supplementary Table S4**). The KEGG results indicate that clinical isolates exhibit differentially enriched pathways, including transcription factors and bacterial secretion systems, compared to environmental isolates. In contrast, environmental isolates show distinct pathway enrichment, glycosyltransferases and lipopolysaccharide biosynthesis proteins, relative to clinical isolates (**Figure 5A, B**). We further analyzed the differentially distributed genes by mapping them to the COG database. Among these, only 40 genes could be mapped to COG categories, with 18 genes showing a bias toward clinical isolates and 22 genes exhibiting a preference for environmental isolates (**Supplementary Table S5**). However, no significant differences were observed in the COG functional annotations of these genes between clinical and environmental isolates (**Figures 5C, D**).

### 3.5 Mobile genetic element analysis

**Figure 6:**
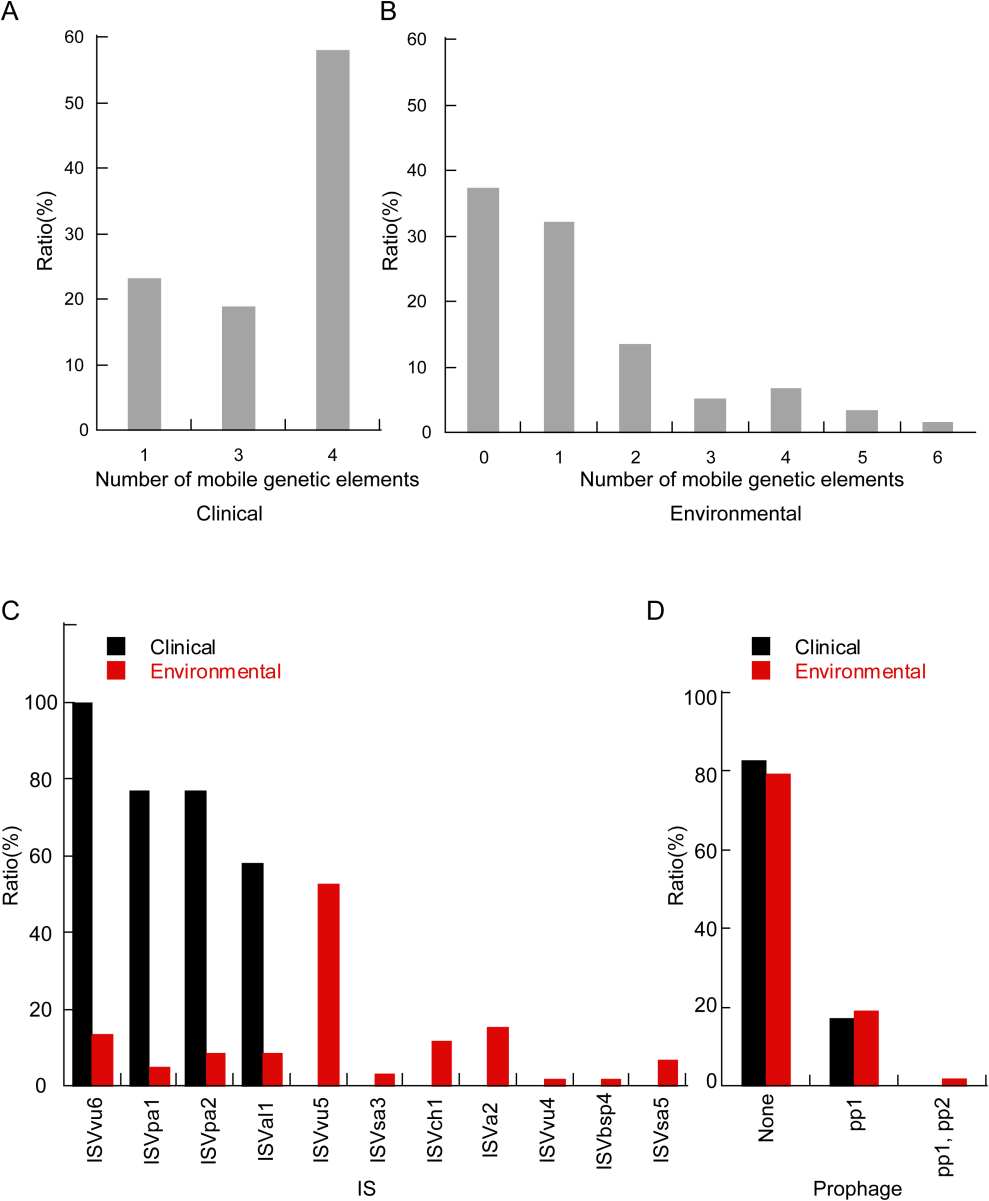
The histogram delineates the distribution of mobile genetic elements between clinical and environmental isolates. The proportion of different numbers of mobile gene elements in clinical **(A)** and environmental **(B)** isolates. **(C)** Comparative distribution of insertion sequence types within clinical and environmental isolates. **(D)** Proportional distribution of distinct prophage between clinical and environmental isolates.

The pan-genomic results indicate significant genetic differences between clinical isolates and environmental isolates. Consequently, we characterized the genome plasticity by evaluating mobile gene elements (MGEs). Many MGEs also carry accessory genes. The benefits of accessory gene carriage can be seen in the success of integrons, elements first identified on MGEs that appear adapted for the acquisition, assembly, and expression of accessory genes (Partridge et al., 2009; Lacotte et al., 2017). In this study, we identified insertion sequences and prophages within the isolates. Clinical isolates contained one, three, or four insertion sequences, with over half carrying four such sequences (**Figure 6A**). Environmental isolates harbored between 0 to 6 insertion sequences (**Figure 6B**). Specifically, four insertion sequences—namely ISVvu6, ISVpa1, ISVpa2, and ISVal1—were identified in clinical isolates. In contrast, environmental isolates exhibited 11 different insertion sequences, with ISVvu5 being the most frequently detected (**Figure 6C**).

Regarding prophages, 17% of clinical isolates were found to carry the pp1 prophage, whereas approximately 19% of environmental isolates contained pp1. Additionally, 2% of the isolates carried both pp1 and the pp2 prophage (**Figure 6D**).

## 4 Discussion

This study found that the age group with the highest infection rate of *V. parahaemolyticus* was between 18 to 40 years old (**Figure 1**), which was consistent with previous research (Wang et al., 2021). Individuals over the age of 66 had a lower risk of infection, aligning with previous research findings (Gong et al., 2018; Wang et al., 2021). The diversity in age distribution may reflect natural changes in host immunity (Simon et al., 2015) and alterations in dietary habits associated with aging. Additionally, the primary symptoms reported by patients treated in Huzhou First People’s Hospital include abdominal pain, diarrhea, nausea, and vomiting. In severe cases, systemic symptoms such as fever and thirst occur, which is consistent with other research findings (Qi et al., 2016). However, the incidence of fever and dehydration is relatively low (**Figure 1**). Furthermore, we found that most patients infected with the O4:KUT serotype exhibit more severe clinical manifestations (**Figures 1**, **2**). TCP, composed of *tcpA*, is a toxin-coregulated pilus that mediates the intestinal colonization of *V. cholerae (* Taylor et al., 1987). *wbfU* was identified in clinical, shrimp-pathogenic strains but were absent in non-pathogens (Xue et al., 2023). The presence of *tcpA* and *wbfU* may enhance the virulence of O4:KUT serotype, although the underlying molecular mechanisms require further investigation. Additionally, the regulatory roles of *wbfY* and *wecA* in bacterial pathogenicity remain to be elucidated. This serotype first appeared in Zhejiang province in 2013 and was the most dominant that year (Zhu et al., 2020). According to our long-term monitoring results, *V. parahaemolyticus* O4:KUT has been a local dominant serotype since 2017 (Zhang et al., 2022). Our previous research has identified at least five ST types within the O4:KUT serotype (Zhang et al., 2024). Nearly all O4:KUT isolates in this study are associated with more severe symptoms (**Figure 2**). In 2021, the positive strains of *V. parahaemolyticus* in Huzhou were almost exclusively O10:K4, leading to a relative decrease in other serotypes, including O4:KUT. Moreover, the number of infections from May to October each year between 2020 and 2023 was significantly higher than that from November to April (**Figure 1**). This pattern aligns with previous research (Lu et al., 2020), which suggests that warmer seasons are more conducive to contracting *V. parahaemolyticus*.

Using the Orthofinder software, we detected a total of 13,003 orthogroups and found that environmental isolates had a higher proportion of orthogroups belonging to the core orthogroups than clinical isolates. A smaller proportion belonged to unique orthogroups (**Figure 3**). Bacteria frequently encounter numerous environmental abiotic stresses (heat, cold, osmotic, salt, oxidation, pH, and radiation) and biotic stresses (antimicrobial compounds and microbial toxins) in their natural life cycle (Beales, 2004), and a greater number of core orthogroups may be necessary for survival under such complex environmental pressures. The PEPPAN software analysis revealed 15,783 genes among these 128 isolates, which exceeds the pan-genome size reported in previous studies (Meparambu Prabhakaran et al., 2022). This increase is attributed to the higher number of *V. parahaemolyticus* isolates included in this study, suggesting that the pan-genomic gene count rises with an increase in the quantity of isolates. However, the difference in the number of core genes, 3,712, compared to previous studies is relatively small, indicating that the core genome is relatively stable. On average, *V. parahaemolyticus* possesses 4,630 genes (Meparambu Prabhakaran et al., 2022), with core genes constituting over 80% of this total. We found *atpB*, *sctC*, *yscU*, *ISVsa5*, *ISVch8*, *ssaV*, *yopJ*, *spaP*, *pdeL* and *tdh2* were exclusively distributed in clinical isolates (**Figure 4**). YscU, SctC, SsaV, YopJ are secreted proteins or effector proteins associated with the type III secretion system (Login and Wolf-Watz, 2015; Ma and Ma, 2016; Yu et al., 2018), while SpaP is involved in bacterial adhesion (Ma et al., 1991). PdeL is a cyclic di-GMP phosphodiesterase. The bacterial second messenger, c-di-GMP, regulates the formation, movement, cell cycle progression, development, and virulence of bacterial biofilms (Hengge, 2016). Thermostable direct hemolysin (TDH) is a proteinaceous toxin that is considered a major virulence factor associated with the ability of *V. parahaemolyticus* strains to cause foodborne gastroenteritis (Shimohata and Takahashi, 2010; Letchumanan et al., 2014). The *tdh2* gene exhibits 97.2% homology with *tdh1* and has been identified as primarily responsible for the phenotypic expression of hemolytic activity (Bhowmik et al., 2014). Therefore, most of these genes, which are exclusively found in clinical isolates, are associated with virulence.

KEGG pathway enrichment analyses have shown differences between clinical isolates and environmental isolates. Both clinical and environmental isolates share secretion system, transporters, prokaryotic defense system, bacterial motility proteins and biofilm formation (**Figure 5**). The protein secretion system is crucial for bacterial growth and is involved in a variety of processes. Almost all bacteria possess some secretion systems that secrete various substrates (Green and Mecsas, 2016). The KEGG annotation results indicate that clinical and environmental isolates protect themselves through prokaryotic defense systems. These prokaryotic defense systems can be classified into two broad groups, differing in their modes of action. The first group consists of defense systems that operate on the principle of self-non-self discrimination, with DNA typically being the target of discriminatory recognition; these mechanisms can be considered as forms of prokaryotic immunity (Makarova et al., 2013). Additionally, within this group is the DNA phosphorothioation system (known as the DND system), which marks DNA through phosphorothioation and degrades unmodified DNA (He et al., 2007; Liang et al., 2007; Xu et al., 2010). The ability of *Vibrio* spp. to adapt to and survive within eukaryotic hosts, as well as to endure varying aquatic environmental conditions, is largely attributed to their capacity to form biofilms (Yildiz and Visick, 2009; Lutz et al., 2013). A biofilm is a structured community of bacterial cells embedded within a self-produced matrix of extracellular polymeric substances (EPS). This matrix facilitates nutrient acquisition and provides a protective barrier against the penetration of antimicrobial agents (Hathroubi et al., 2017). Previous studies showed that the flagellar motility plays an important role in the formation of biofilm in the life cycle of vibrios (Echazarreta and Klose, 2019). Lipopolysaccharide biosynthesis is one pathway that is enriched in environmental isolates. Lipopolysaccharide (LPS) is a type of glycolipid found in the outer leaflet of the outer membrane (OM) of Gram-negative bacteria (Muhlradt and Golecki, 1975; Kamio and Nikaido, 1976). The presence of LPS on the cell surface enhances the barrier function of the OM, which renders many antibiotics ineffective that are typically used to treat infections caused by Gram-positive pathogens (Nikaido, 2003). Numerous transcription factors orchestrate the spatiotemporal regulation of virulence factors in *Vibrio vulnificus*, integrating diverse environmental cues such as nutrient availability, bacterial cell density, and the presence of antimicrobial agents (Choi and Choi, 2022). Previous study suggested that transcription factor CytR plays an important role in *V. cholerae* pathogenesis (Das et al., 2020). Therefore, the regulatory pathways mediated by transcription factors in clinical isolates, as opposed to environmental isolates, may play a critical role in determining their pathogenicity. Furthermore, a significant number of genes remain unmatched to KO numbers, and further research is needed to elucidate the enriched pathways associated with these genes.

Previous studies have shown that among these 128 isolates, environmental isolates carry a more diverse array of resistance genes compared to clinical isolates (Zhang et al., 2024). The majority of AMR-encoding genes in *V. parahaemolyticus* are primarily acquired through horizontal gene transfer (HGT) (Pazhani et al., 2021). This study indicates that a greater variety of mobile genetic elements can be detected in environmental isolates, and a higher proportion of these isolates harbor prophages (**Figure 6**). Insertion sequence (IS) elements are a class of small mobile genetic elements that are ubiquitously distributed across the genomes of most bacterial species. Accumulating evidence indicates that IS elements play a significant role in mediating insertion mutations, facilitating genome rearrangements, promoting the dissemination of antibiotic resistance genes and virulence factors both within and between species, and modulating the expression of neighboring genes (Mahillon and Chandler, 1998). Previous studies have demonstrated that IS*Vpa2* exhibits the ability to insert at multiple genomic sites. Furthermore, IS*Vpa2* has been shown to induce genetic rearrangements, including insertional inactivation of target genes and adjacent deletions. Additionally, IS*Vpa2* can be horizontally transferred among species within the genus *Vibrio (* Kamruzzaman and Nishibuchi, 2008). The transfer of MGEs between different bacteria is facilitated by HGT. As a major force in microbial evolution, HGT enables the acquisition of novel functions on a large scale and allows for rapid adaptation to new niches (Wiedenbeck and Cohan, 2011). Previous research has shown that HGT can significantly drive adaptation in food systems and other environments managed by humans (Rossi et al., 2014; Akanni et al., 2015). Previous studies have revealed that IS*Val1* facilitates the HGT of prophages, plasmids, and genomic islands among *V. parahaemolyticus* populations in response to adverse environmental conditions and selective pressures. Additionally, IS*Val1* has been shown to mediate the formation of MGEs associated with antibiotic resistance (Fu et al., 2021). IS*Vsa3* has been identified as being strongly associated with MDR in *Salmonella* (Lewis et al., 2023). IS*Vsa5* has been shown to play a crucial role in modulating the expression of *bla*_CTX-M_ in *E. coli* (Black et al., 2024). Further investigation is required to elucidate the roles and mechanisms of other mobile genetic elements (MGEs). Therefore, we hypothesize that the elevated concentration of mobile genetic elements (MGEs) in environmental isolates contributes to enhanced environmental adaptation.

In summary, statistical analysis of patients treated at the First People’s Hospital of our city over recent years has revealed that the primary population affected by *V. parahaemolyticus* is aged 18-40, with a higher infection rate observed during warmer seasons. Analysis of infection characteristics revealed that patients infected with the O4:KUT serotype exhibited more severe symptoms. Although clinical isolates had a smaller number of serotypes, their core orthogroups still accounted for a smaller proportion compared to environmental isolates. Pan-genomic analysis results indicated that there was a significant difference in gene expression between clinical and environmental isolates, which may be attributed to the higher presence of mobile genetic elements (MGEs) in environmental isolates.

## Data Availability

The original contributions presented in the study are included in the article/**Supplementary Material**. Further inquiries can be directed to the corresponding author.
