# Supplementary material for: Comparative pan-genomic analysis reveals pathogenic mechanisms and genomic plasticity in Vibrio parahaemolyticus clinical and environmental isolates
Source: Front Cell Infect Microbiol. 2025 Apr 10;15:1574627. doi: 10.3389/fcimb.2025.1574627 (PMC12018335; doi:10.3389/fcimb.2025.1574627)
Supplement: Supplementary file 1 [file DataSheet1.docx]

Supplementary Material


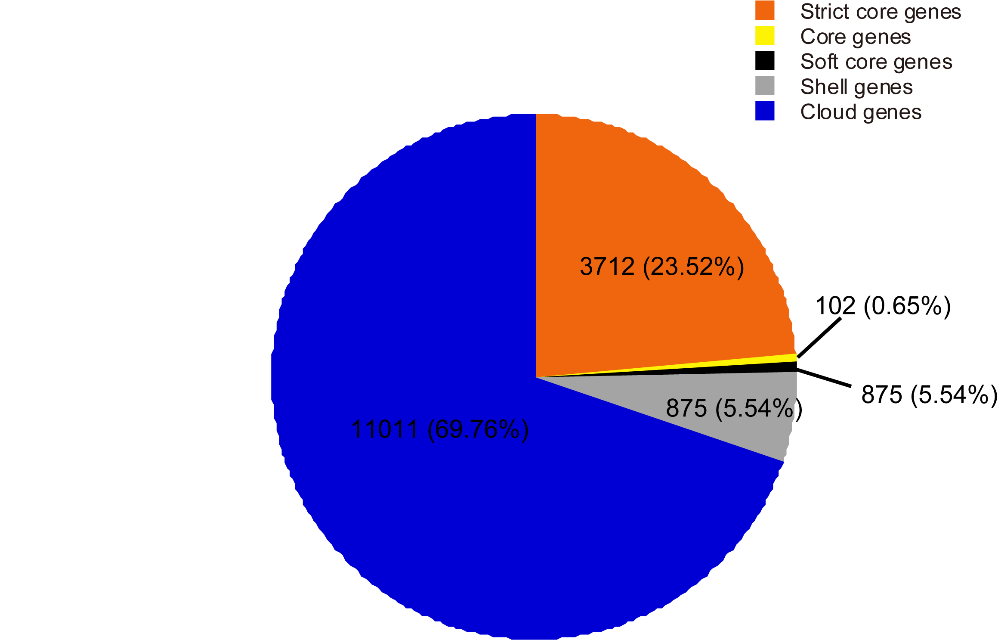


**Supplementary Figure 1.** Pie chart illustrating the pan-genome composition of 128 *V. parahaemolyticus* isolates. Values indicate both absolute counts and relative percentages of strict core (present in all isolates), core (present in 99%-100% isolates), soft core (present in 95%-99% isolates), shell (present in 15%-95% isolates) and cloud genes (present in 0%-15% isolates).
